# Maternal Transmission Effect of a *PDGF-C* SNP on Nonsyndromic Cleft Lip with or without Palate from a Chinese Population

**DOI:** 10.1371/journal.pone.0046477

**Published:** 2012-09-28

**Authors:** Di Wu, Mei Wang, Xingang Wang, Ningbei Yin, Tao Song, Haidong Li, Feng Zhang, Yongbiao Zhang, Zhenqing Ye, Jun Yu, Duen-Mei Wang, Zhenmin Zhao

**Affiliations:** 1 Department of Cleft Lip and Palate, Plastic Surgery Hospital, Chinese Academy of Medical Sciences and Peking Union Medical College, Beijing, People's Republic of China; 2 Chinese Academy of Sciences Key Laboratory of Genome Sciences and Information, Beijing Institute of Genomics, Chinese Academy of Sciences, Beijing, People's Republic of China; 3 Graduate University of Chinese Academy of Sciences, Beijing, People's Republic of China; 4 Department of Stomatology, Beijing Children Hospital Affiliated to Capital University of Medical Sciences, Beijing, People's Republic of China; 5 People's Hospital of Jincheng City, Jincheng, Shanxi, People's Republic of China; National Institute of Environmental Health Sciences, United States of America

## Abstract

Cleft lip with or without palate (CL/P) is a common congenital anomaly with a high birth prevalence in China. Based on a previous linkage signal of nonsyndromic CL/P (NSCL/P) on the chromosomal region 4q31–q32 from the Chinese populations, we screened the 4q31–q32 region for susceptibility genes in 214 trios of Han Chinese. *PDGF-C*, an important developmental factor, resides in the region and has been implicated in NSCL/P. However, in our family-based association test (transmission disequilibrium test; TDT), we could not conclude an association between *PDGF-C* and NSCL/P as previously suggested. Instead, we found strong evidence for parent-of-origin effect at a *PDGF-C* SNP, rs17035464, by a likelihood ratio test (unadjusted p-value = 0.0018; *I_m_* = 2.46). The location of rs17035464 is 13 kb downstream of a previously reported, NSCL/P-associated SNP, rs28999109. Furthermore, a patient from our sample trios was observed with a maternal segmental uniparental isodisomy (UPD) in a region containing rs17035464. Our findings support the involvement of *PDGF-C* in the development of oral clefts; moreover, the UPD case report contributes to the collective knowledge of rare variants in the human genome.

## Introduction

Cleft lip with or without palate (CL/P) is one of the most common birth defects, affecting 0.5–1% of newborns worldwide [1], [2]. Nonsyndromic CL/P (NSCL/P) accounts for ∼70% of cases with cleft lip with or without palate, and is genetically distinct from those with syndromic CL/P [3]. NSCL/P has heterogenic etiologies including several susceptibility genes and environmental risk factors [4], [5], [6], [7]. With human linkage studies, several susceptibility loci of NSCL/P have been located to the regions of *MTHFR*, *TGFA*, 4q28 (D4S175), *F13A1*, *TGFB3*, 17q12 (D17S250) and *APOC2*. Genes found in the susceptibility loci are involved in a wide range of cellular functions, such as growth promotion, transcriptional regulation, xenobiotic metabolism, nutrient metabolism, and immune response [8].

The first genome-wide linkage scan of NSCL/P in Chinese families found the most significant signal to be at chromosome 4q31–q32 [9]. Other NSCL/P studies reported linkage signals involving 4q31–q32 in Filipino and Syrian families [10], [11]. The involvement of chromosome 4q31–q32 in human CL/P was also suggested by both cytogenetic deletion and association studies [12], [13], [14], [15], [16]. The rs28999109 SNP found to associate with NSCL/P in Chinese families was mapped to the regulatory region of platelet derived growth factor C (*PDGF-C*, [MIM:608452]) at 4q32 [17]. The role of *PDGF-C* in palatogenesis was also supported by a mouse knockout study in which the *Pdgfc* (−/−) embryos had a phenotype of cleft face [18].

Without considering the parental origin of an allele, association analysis is unable to distinguish the parental preference of transmission on a disease variant. The imprinting process provides a mechanism for a pair of parental alleles to be functionally unequal. A recent association study of type 2 diabetes demonstrated that the parent-of-origin effects had been overlooked by the past large-scale genome-wide association studies [19]. With our family-based study design, the parent-of-origin effects could be taken into consideration.

Uniparental disomy (UPD) is another genetic mechanism that distorts Mendelian transmission. UPD occurs when a child receives both copies of a chromosome or chromosomal region from only one parent, leading to the violation of biparental inheritance [20]. Depending on the genomic location and size of the affected region, UPD can be clinically undetectable. However, abnormal expression of critical developmental genes due to UPD may lead to harmful phenotypes. Thus far, no studies have suggested UPD as one of the mechanisms in CL/P formation.

In this study, a two-step fine mapping approach was used to locate the susceptibility genes of NSCL/P at the 4q31–q32 region in Han Chinese. We genotyped 10 STR (Short Tandem Repeat) markers in 214 NSCL/P trios and subsequently fine mapped with SNP markers in a subset of our subjects. Later, the parent-of-origin effect of candidate genes was assessed by a parent-of-origin likelihood ratio test (PO-LRT).

## Materials and Methods

### Study Subjects

We collected 214 case-parent trios through the CL/P Treatment Center, Plastic Surgery Hospital, Chinese Academy of Medical Sciences. All probands underwent clinical genetics evaluations to exclude congenital anomalies other than CL/P and any major developmental delay, and were diagnosed as an isolated, nonsyndromic CL/P. Patients with cleft palate only were not included in this study. The probands consisted of 156 boys and 58 girls with an approximately 3∶1 ratio between males and females. In addition, 24 of the 214 trios had positive family history of CL/P. Table 1 is a summary of our sample information. Written informed consents were obtained from all probands' parents. The study was approved by the Ethical Institutional Committee of the Plastic Surgery Hospital, Chinese Academy of Medical Sciences.

**Table 1:** Description of sample in two-step genotyping.

Number of trios	Step 1	Step 2
Total	214	160
Boys vs. girls in probands	156∶58	115∶45
Triads vs. Duos	205∶9	160∶0
Trios have family history or not	22∶192	14∶146

### Marker Selection

A two-step strategy was used to map the NSCL/P susceptibility gene at the signal, 4q31–132, previously reported in a genome-wide linkage study [21]. First, we genotyped the 4q31–q32 region with 10 STR markers from D4S1644 to D4S2431 (141 Mb–174 Mb, spanning 30 Mb) of the deCODE genetics map [22]. The 10 STR markers were chosen for their high heterozygosity and long PCR product to increase their information content and raise the success rate of genotyping (Table S1). Our complete sample set, which consisted of 214 trios, was genotyped during this step. Second, the target region was shortened to a 500-kb region including D4S413 and the *PDGF-C* promoter. The location of the 500-kb region was based on two evidences: 1) we observed the lowest p-value at D4S413 in our TDT analysis of the first step; also, 2) Choi et al. [17] reported an association signal to NSCL/P at the *PDGF-C* promoter. To investigate the transmission disequilibrium and possible small genomic changes, we mapped the 500-kb region with 80 SNP markers (rs894588–rs17036150) from HapMap database, roughly spaced at 5 kb/SNP (Table S2). To be cost-effective, 160 of the 214 trios having more severe clinical symptoms were subjected to the second step of genotyping. Our clinical criteria for the severity of cleft lip and cleft palate were adapted from a guideline used by Song et al. [23]. Cleft lip was graded as “microform” for the vermilion only, “moderate” when restricted to the lip, and “severe” when extending from the vermilion free border to the nasal floor. Likewise, cleft palate was graded as “microform” for the uvula only, “moderate” when restricted to the posterior of the incisive foramen, and “severe” when encompassing the entire hard and soft palate (beyond the incisive foramen).

### Genotyping

All DNA samples were extracted from whole blood using the QIAamp DNA Mini Kit (Qiagen, USA). For STR genotyping, one of each primer pairs was 5′ FAM-labeled (Invitrogen, Shanghai, China), and PCRs were performed on the GeneAmp PCR system 9700 (Applied Biosystems, USA). Electrophoresis of the amplified products was conducted on an ABI 3730 XL DNA Analyzer (Applied Biosystems, USA). A size-call panel was trained for each marker, using the software GeneMarker (SoftGenetics, USA), with data from a random successfully typed 96-well plate. Fragment sizes of a reaction were automatically determined with established panels and manually double-checked by two readers. Unsuccessful reactions were retried until either success or three failures.

The SNP genotyping was performed using Illumina VeraCode® technology on the BeadXpress® system (Illumina, USA). Genotypes were generated on its accompanying GenomeStudio software suite (version 1.0). GenCall scores <0.25 at any locus were considered “no calls”. All 80 SNPs succeeded, and overall genotype call rate was 98%.

For all genotyped markers and trios, Mendelian consistency was checked within each trio family and inconsistency was found in family 152. To further investigate the issue, a genome-wide genotyping was conducted on the proband of family 152 with the Illumina Human660W-Quad v1 DNA Analysis BeadChip according to the manufacturer's specifications (Illumina, USA). GenCall scores <0.15 at any locus were considered “no calls”. The genotype call rate is 99%. Image data was analyzed using the Chromosome Viewer tool contained in GenomeStudio (Illumina, USA). All genomic positions were based upon NCBI Build 36 (dbSNP version 126).

### Statistical Analyses

For the STR markers, the rarest alleles were combined to generate a pseudo-allele until it reached a threshold of 0.1. Hardy-Weinberg equilibrium test was performed by the gap package in R [24]. The allele frequency and Hardy-Weinberg equilibrium test of SNP markers were computed from parents using the Haploview program [25] with, a cutoff of 0.05. Pairwise linkage disequilibrium (LD) was evaluated with r^2^ for all SNPs using the Haploview program.

The transmission disequilibrium test (TDT) was performed using the family-based association test (FBAT) program [26]. Haplotype-based TDT analysis of SNP markers in the second step was performed by the Haploview program. Haplotype blocks were constructed based on the default algorithm [27] in Haploview. The correction for multiple testing on haplotype-based TDT was conducted with 1000-permutations in Haploview. Our first step genotyping used only 10 STR markers, and the multiple testing was adjusted with Bonferroni correction. For the second step genotyping, we employed more closely spaced markers with increasing possibility of forming background LD among markers. Therefore, we adjusted the p-values in our second-step analysis with Li's method [28] from the SNPSpD software [29]. The method uses the pairwise LD values between SNPs for spectral decomposition (SpD) matrices.

To test the parent-of-origin effect, a parent-of-origin likelihood ratio test (PO-LRT), implemented in the LEM software, was used to estimate the relative risks for heterozygotes and homozygotes of overtransmissed alleles. The PO-LRT model considers the inherited number of copies of the overtransmissed allele as maternal genotypic effects on the phenotype of a fetus [30]. The estimated risk ratios (*I_(m)_*) for an imprinting effect suggest that there is an excess maternal transmission compared to paternal transmission in this region when *I*_(m)_>1, and vice versa.

## Results

### TDT and Parent-of-Origin Effects

Our first step genotyping with 10 STR markers was on a 30-Mb region encompassing the linkage peak reported by Marazita at 4q31-q32 [9]. The heterozygosity of the STR markers estimated from the parents is displayed in Table S1. All of the markers passed the Hardy-Weinberg equilibrium test. With our complete NSCL/P sample set of 214 trios, we found most-significant association between the D4S413 marker and the disease by TDT (unadjusted p-value = 0.01974). However, after stringent adjustment for multiple testing with the Bonferroni correction, the p-value of this association did not reach the significance level of 0.05. In addition, we observed a non-Mendelian transmission of D4S1498 in family 152, for the patient received both copies of D4S1498 only from the mother.

**Figure 1:**
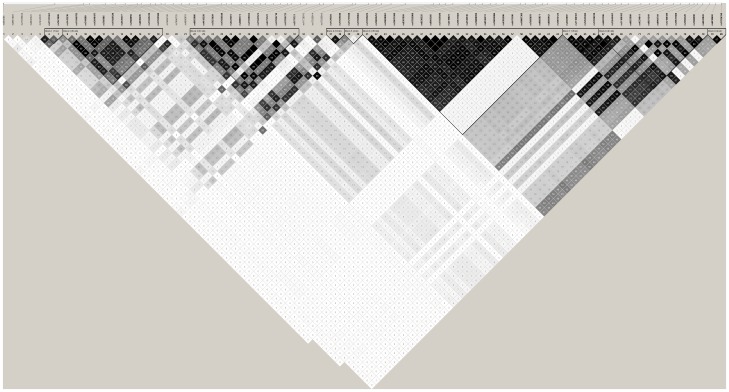
Linkage disequilibrium (LD) pattern of the 80 SNPs in step 2. Linkage disequilibrium derived from the parents of our sample set as measured in *r* ^2^. White, *r* ^2^ = 0; Shades of gray, 0<*r* ^2^<1; Black, *r* ^2^ = 1.

In the second step genotyping, the minor allele frequencies of the SNP markers estimated from the parents are displayed in Table S2. All of the markers passed the Hardy-Weinberg equilibrium test. Figure 1 shows that the LD pattern among the 80 SNPs. Individually testing each SNP by TDT analysis, we found the lowest unadjusted p-value of 0.0066 at SNP rs10517665. To correct for the presence of LD, we calculated the effective number of the 80 SNPs employing Li's method [28] and used the value, 24, for correction of multiple testing. However, no SNPs reached the significant level after correction with the effective number for multiple testing. The result is summarized in Table S3. Ten haplotype blocks were identified based on LD. We observed the lowest p-value (0.0043; 0.06 after correction) on the haplotype GTGAAGCAAAGA, consisting of 12 SNPs from rs4691396 to rs6843849. No haplotypes are statistical significant after 1000-permutations. The result of haplotype-based TDT analysis is presented in Table S5.

**Figure 2:**
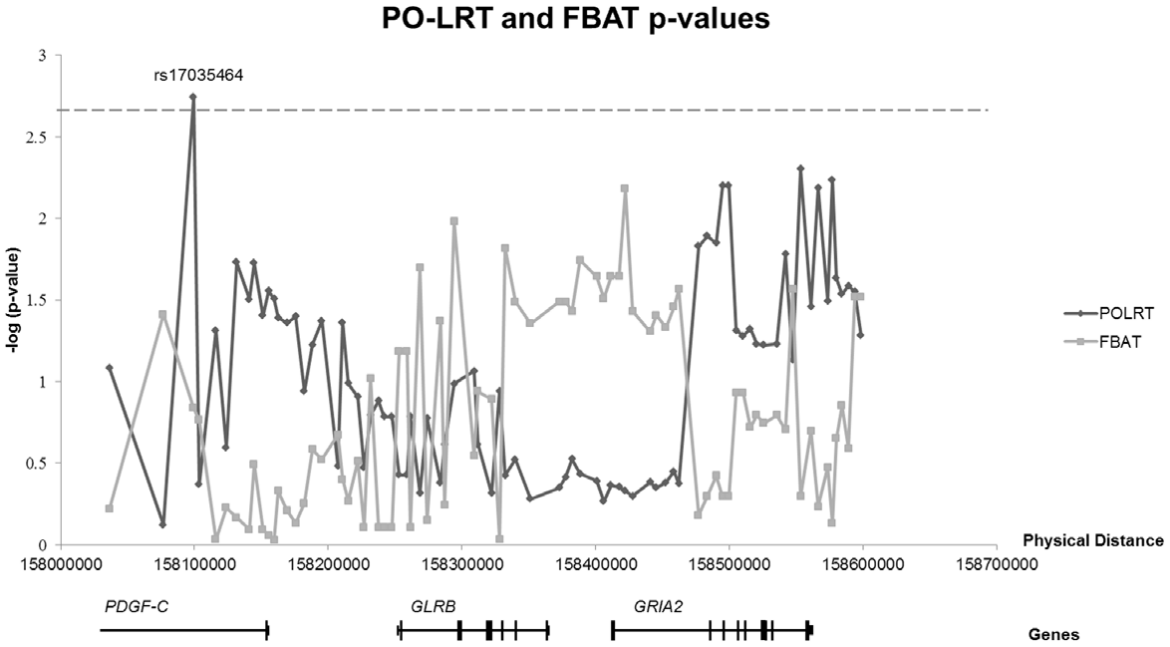
Scatter diagram of the p-values of FBAT and PLORT tests. Scatter diagram of the p-values (−log_10_ (p-value)) of FBAT and POLRT tests. The p-value of rs17035464 exceeds the corrected threshold of multiple testing.

Considering the parent-of-origin effects, we found an excess of maternal transmission and a deficit of paternal transmission of the allele A of rs17035464 (unadjusted p-value = 0.0018, *I*_(m)_ = 2.46) in *PDGF-C* (Figure 2). After correction with the effective number of our SNPs, the adjusted p-value (0.0432) remained significant. Table S3 shows the result of the PO-LRT test.

### Evidence of UPD

**Figure 3:**
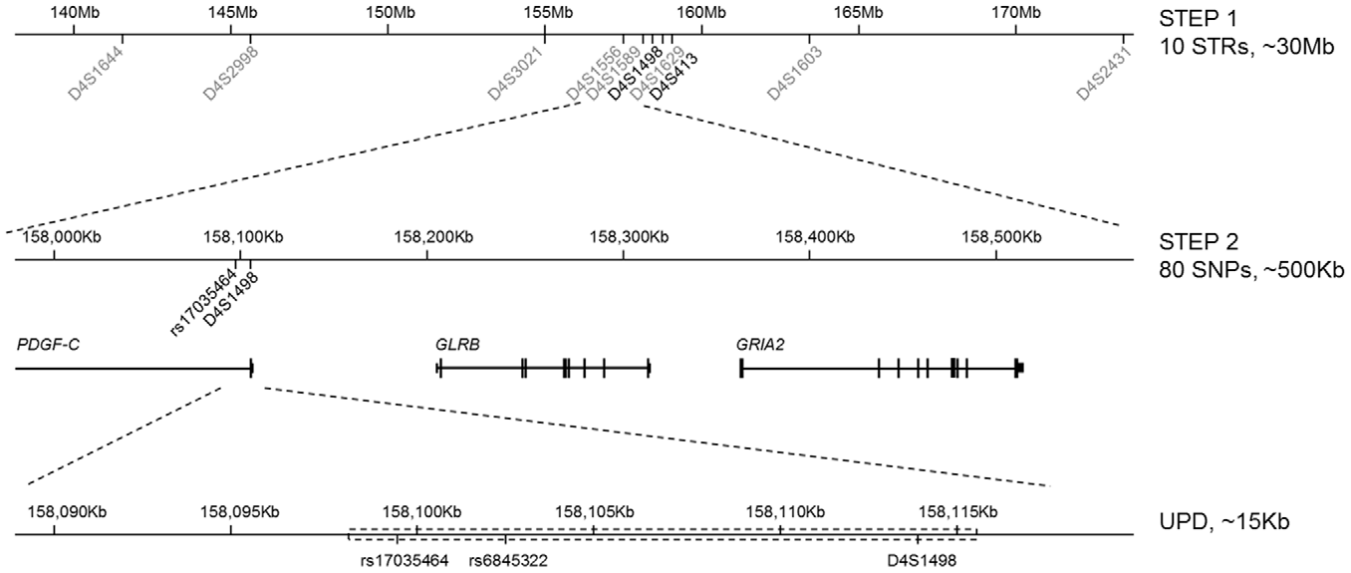
Schematic diagram of two-step mapping strategy. The genotyping was first conducted with 10 STR markers covering a 30-Mb region on 4q31–q32 and followed by 80 SNPs spanning the region from D4S1498 to D4S413. Lastly, the minimal UPD region was estimated to be from rs17035464 to D4S1498 in a physical distance of 14 Kb.

After genotyping, an aberrant transmission of D4S1498 was found in family 152. Proband 152 obtained both alleles of D4S1498 from the mother, but none from the father (Table S4). We then examined the markers surrounding D4S1498 for Mendelian inconsistence. Two SNPs, rs17035464 and rs6845322, that are about 10 kb away from D4S1498 (Figure 3) transmitted in the same pattern as D4S1498. All genotypes of the other STR and SNP markers followed the Mendelian transmission. Whole genome genotyping and CNV analysis were performed on proband 152 and indicated that the region containing rs17035464, rs6845322, and D4S1498 was diploid in content, which means no deletion at the region (Figure S1). Taken together, the abnormal transmission of a minimal region of 14 kb, from rs17035464 to D4S1498, is likely to be a result of segmental maternal uniparental isodisomy (UPD), and the region is mapped to the *PDGF-C* promoter.

Proband 152 was a 1-year-old girl with bilateral cleft lip and palate, and no other noticeable abnormalities were found after a comprehensive postnatal physical examination (Figure 3). The proband is the first child from a healthy, non-consanguineous pair of parents. No history of any other family members with a cleft lip or other congenital defects was reported. Furthermore, neither passive smoking nor folic acid supplementation during the first trimester was reported.

## Discussion

In this family-based association study, we aimed to fine-map the susceptibility genes of NSCL/P on 4q31–q32 with 214 Chinese trios. Our selection of the 4q31–q32 region was based on a previous linkage signal from a genome-wide study with Chinese families by Marazita [9]. Their highest multipoint linkage peak fell at the STR marker, D4S1629, on 4q31–q32. Although we genotyped the region with 10 STRs and 80 SNPs in a two-step approach, without considering the parent-of-origin effects, no markers reached the significant level after correction for multiple testing.

During the first step of the approach, we genotyped the 4q31–q32 region with 10 STR markers and found the lowest p-value at D4S413, which is in strong LD with the previously reported D4S1629. Both of the STR markers were located upstream on the *GRIA2* gene. During the second step of genotyping, we found the lowest p-value of our individual SNP tests at rs10517665 in *GRIA2*. A haplotype analysis of TDT showed that the GTGAAGCAAAGA haplotype with the lowest p-value is also located upstream in *GRIA2*. The GTGAAGCAAAGA haplotype contains several SNPs from rs4691396 (Chr4:158525561) to rs6843849 (Chr4:158588880). Interestingly, this region contains the D4S413 and D4S1629 markers. Together, these lines of evidence indicate an involvement of *GRIA2* in NSCL/P. The gene product of *GRIA2* is a member of the glutamate receptor family. This family is sensitive to alpha-amino-3-hydroxy-5-methyl-4-isoxazole propionate (AMPA) and functions as ligand-activated cation channels. Glutamate receptors play an important role in excitatory neurotransmission, with no known biological functions related to embryonic development [31].

From a subset of Chinese families used in Marazita's study of NSCL/P, Choi reported an association between NSCL/P and the *PDGF-C* SNP rs28999109 [17]. This NSCL/P-associated SNP is located in the promoter region of *PDGF-C*. Without considering the parent-of-origin effects, our study did not yield any significant association between NSCL/P and *PDGF-C*. However, we observed low p-values from our association test in *GRIA2*. The *GRIA2* gene is located next to *PDGF-C* with a physical distance of 240 kb, and their transcriptional units face away from each other. This 240-kb intergenic region of *GRIA2* and *PDGF-C* contains a number of highly conserved segments, which may influence the simultaneous expression of both genes. Furthermore, in a genome-wide study, Pickrell et al. reported signals of positive selection on *GRIA2* and another nearby gene, *GLRB*, in an Asian-specific manner [32]. Although based on limited observation, it is possible that the weak association that we noticed between *GRIA2* and NSCL/P may be due to the nearby *PDGF-C* gene.

After including the parent-of-origin effects in our TDT analysis, we observed a maternally over-transmitted allele A on rs17035464, located in the *PDGF-C* promoter. Other than Choi's report, there are numerous lines of evidence to support the role of *PDGF-C* in palate formation. The *Pdgfc* knockout mice have a cleft face phenotype [33], [34], indicating an important role of *PDGF-C* in the fusion of palate shelf during embryonic growth. Also, a zebrafish study demonstrated that Pdgf signaling was negatively regulated by binding of microRNA Mirn140 to *Pdgfra* 3′ UTR during palatal development [35]. Other animal studies reported that both Pdgfa and Pdgfc can regulate the development of palate via binding to Pdgfr-α.

The *PDGF-C* gene is a newly found member of the *PDGF* family. The encoded product, PDGF-CC, binds to the PDGF receptors (PDGFR-αα and PDGFR-αβ) and subsequently activates the extracellular signal-regulated protein kinase/mitogen-activated protein kinase (Erk/MAPK) and Akt/PKB pathways by phosphorylation [36], [37], [38]. Through these two signaling pathways, *PDGF-C* regulates the processes of cell proliferation, survival, and migration, as well as deposition/maintenance of extracellular matrix [39]. Parent-of-origin effects have been previously reported for several CL/P candidate genes, namely *MTHFR*, *TGFA*, and *RUNX2* [40], [41], [42]. The mechanism of parent-of-origin effects remains elusive. One potential mechanism is mediated through genomic imprinting. To our knowledge, *PDGF-C* has not been reported as an imprinted gene. However, a CpG island (Chr4: 158112136–158112736) can be found adjacent to the *PDGF-C* SNP rs17035464 and also resides within the segmental UPD region of proband 152 (Figure 3). Despite any biological evidence here, it is still possible that failed imprinting process at the region may disturb embryonic and neonatal growth, such as found in Prader-Willi syndrome. A direct relationship between CL/P and imprinting has not been reported, and the imprinting study of *PDGF-C* genomic region may provide clues to the causal effect of the gene on NSCL/P.

In addition to the association analysis, we used genotyping information of a trio family for alleles violating Mendelian transmission. Our proband 152, a 1-year-old girl with bilateral cleft lip and palate, was found to contain a segmental maternal uniparental isodisomy on 4q31–q32 [25], [43], [44]. All our marker information on 4q31–q32 were used to validate the aberrant transmission on D4S1498 in family 152. Three contiguous markers, rs17035464, rs6845322, and D4S1498, were found with both alleles of the proband exclusively derived from the mother. The three markers constitute a minimal region of 14 kb in question (Figure 3). The rest of the markers were either transmitted in the Mendelian mode or uninformative. To define the largest region, two informative SNPs, rs894588 (Chr4: 158036593) and rs7674099 (Chr4: 158131475), marked the boundaries for the Mendelian transmission, were identified to be 94 kb apart. As a result, the UPD region is between 14 to 94 kb in length and maps to the promoter region of *PDGF-C*. This minimal 14 kb region also contains a known SNP, rs28999109, which was reported to affect the transcriptional activity from the *PDGF-C* promoter [17]. Our PO-LRT signal SNP rs17035464 was one of the uniparental transmitted SNPs. Still, there is no direct evidence indicating that this UPD can directly mediate the formation of CL/P. The most relevant report came from a Malay group, who observed a segmental UPD on chromosome 6 from a NSCL/P boy [45]. Without further investigation, the report did not conclude that UPD was a mechanism of NSCL/P. In the future, more cases of normal karyotype with a segmental UPD should be found with the use of a high resolution SNP genotyping microarray, and then we will obtain a better picture of how UPD influences gene expression and leads to the disruption of embryonic development.

In summary, our study of sporadic NSCL/P case-parent trios from Han Chinese demonstrates an association between *PDGF-C* and NSCL/P only when considering parent-of-origin effects. Additionally, an abnormal transmission pattern found in the promoter region of *PDGF-C* of proband 152 suggests that study of small-sized UPD may become a fruitful area for gene mapping of diseases.

## Supporting Information

Table S1 **STR markers in genotyping Step 1.**
(DOC)

Table S2 **SNP markers in genotyping Step 2.**
(DOC)

Table S3 **TDT and PO-LRT analysis.**
(DOC)

Table S4 **Genotypes of family 152.**
(DOC)

Table S5 **Haplotype TDT Analysis.**
(DOC)

Figure S1 **CNV analysis of proband 152 on 4q32.** The whole genome genotyping and CNV analysis was performed by Illumina Human 660W-Quad v1 DNA Analysis BeadChip.
(TIF)
