# Supplementary material for: Maternal Transmission Effect of a PDGF-C SNP on Nonsyndromic Cleft Lip with or without Palate from a Chinese Population
Source: PLoS One. 2012 Sep 28;7(9):e46477. doi: 10.1371/journal.pone.0046477 (PMC3460900; doi:10.1371/journal.pone.0046477)
Supplement: Table S1 — STR markers in genotyping Step 1. (DOC) [file pone.0046477.s001.doc]

Table S1. STR markers in genotyping Step 1

| Marker Name | Genetic Distance (cM) | Physical Distance (bp) | Forward Primer | Reverse Primer | Tm(℃) | Length of PCR | Heterozygosity |
| --- | --- | --- | --- | --- | --- | --- | --- |
| D4S1644 | 144.53 | 141970903 | TAACATTGACCGCTCCTCTC | CATCCTTCCTGGTCCCTAGT | 59 | 186-206 | 0.62 |
| D4S2998 | 147.45 | 145785642 | AAGTTCTTGGGCCGCAG | TTCTACACCCAGGGGAACC | 56 | 129-173 | 0.82 |
| D4S3021 | 157.21 | 155155280 | ACTGGCCTGATGTGGTGA | GGTGCCTGATAGCCTGAA | 58 | 223-245 | 0.65 |
| D4S1556 | 159.9 | 157673422 | GCCATCAACTAACACAGAAAAA | AAACAGTGAGAGACACAGAGTACAG | 61 | 157-171 | 0.28 |
| D4S1589 | 160.02 | 158005552 | CCACTTTATCTATGAAATGAAGGTT | CGTTGGAAGTATGTGAAATGAT | 60 | 207-217 | 0.49 |
| D4S1498 | 160.3 | 158114042 | ATGGGACTTAAATAAAGGGCA | CCCTTCCAATCTCCTGAGA | 59 | 247-256 | 0.62 |
| D4S1629 | 160.3 | 158556260 | TGGTTCTGCTTTTTCTCTCC | TTTAACAGACAAATGACAAATCTG | 57 | 141-157 | 0.68 |
| D4S413 | 160.3 | 158572604 | <ABI>* | <ABI>* | 55 | 282-334 | 0.61 |
| D4S1603 | 164.77 | 163999458 | CAAATATGTCATTTTATATGCTG | TGGTGTGTCTCTGATGCT | 59 | 190-208 | 0.66 |
| D4S2431 | 176.78 | 175057242 | GACCTTTGCCCTGGATAACT | GCCAAGCACTCTTTTCTTCA | 59 | 234-258 | 0.78 |

Tm. Optimal Annealing Temperature

* We used the primers of D4S413 from the ABI Prism Linkage Mapping Set (v2.5).
