# Supplementary material for: Maternal Transmission Effect of a PDGF-C SNP on Nonsyndromic Cleft Lip with or without Palate from a Chinese Population
Source: PLoS One. 2012 Sep 28;7(9):e46477. doi: 10.1371/journal.pone.0046477 (PMC3460900; doi:10.1371/journal.pone.0046477)
Supplement: Table S2 — SNP markers in genotyping Step 2. (DOC) [file pone.0046477.s002.doc]

Table S2. SNP markers in genotyping Step 2

| SNP_Name | Chr | Physical Distance | Gene | Location | HET | HW pvalue | MAF | MAF_ Hap_Chinese |
| --- | --- | --- | --- | --- | --- | --- | --- | --- |
| rs894588 | 4 | 158036593 | PDGF-C | intron | 0.441 | 0.172 | 0.400 | 0.367 |
| rs6851803 | 4 | 158076643 | PDGF-C | intron | 0.060 | 1.000 | 0.030 | 0.044 |
| rs17035464 | 4 | 158099171 | PDGF-C | intron | 0.183 | 0.876 | 0.105 | 0.078 |
| rs6845322 | 4 | 158103555 | PDGF-C | intron | 0.502 | 0.935 | 0.451 | 0.444 |
| rs1443235 | 4 | 158116000 | PDGF-C | flanking_5UTR | 0.427 | 0.891 | 0.299 | 0.356 |
| rs13117461 | 4 | 158123643 | PDGF-C | flanking_5UTR | 0.404 | 0.880 | 0.288 | 0.330 |
| rs7674099 | 4 | 158131475 | PDGF-C | flanking_5UTR | 0.470 | 0.972 | 0.384 | 0.400 |
| rs1443230 | 4 | 158140930 | PDGF-C | flanking_5UTR | 0.492 | 0.149 | 0.344 | 0.344 |
| rs11945782 | 4 | 158144404 | PDGF-C | flanking_5UTR | 0.392 | 0.140 | 0.234 | 0.270 |
| rs716680 | 4 | 158151138 | PDGF-C | flanking_5UTR | 0.486 | 0.202 | 0.341 | 0.344 |
| rs12649197 | 4 | 158155473 | PDGF-C | flanking_5UTR | 0.489 | 0.174 | 0.343 | 0.359 |
| rs765985 | 4 | 158159671 | PDGF-C | flanking_5UTR | 0.486 | 0.202 | 0.341 | 0.344 |
| rs17035528 | 4 | 158162784 | PDGF-C | flanking_5UTR | 0.394 | 0.324 | 0.244 | 0.244 |
| rs4535377 | 4 | 158169250 | GLRB | flanking_5UTR | 0.457 | 0.536 | 0.324 | 0.311 |
| rs7668059 | 4 | 158175791 | GLRB | flanking_5UTR | 0.463 | 0.328 | 0.321 | 0.311 |
| rs6830495 | 4 | 158182104 | GLRB | flanking_5UTR | 0.470 | 0.188 | 0.318 | 0.311 |
| rs11945439 | 4 | 158188299 | GLRB | flanking_5UTR | 0.368 | 0.333 | 0.222 | 0.227 |
| rs17035590 | 4 | 158195266 | GLRB | flanking_5UTR | 0.362 | 0.212 | 0.213 | 0.233 |
| rs7689138 | 4 | 158207179 | GLRB | flanking_5UTR | 0.206 | 1.000 | 0.116 | 0.089 |
| rs7696725 | 4 | 158210676 | GLRB | flanking_5UTR | 0.364 | 0.633 | 0.227 | - |
| rs4690879 | 4 | 158215162 | GLRB | flanking_5UTR | 0.413 | 1.000 | 0.289 | - |
| rs6852066 | 4 | 158222346 | GLRB | intron | 0.441 | 0.219 | 0.392 | 0.389 |
| rs4615228 | 4 | 158226703 | GLRB | intron | 0.371 | 0.472 | 0.265 | - |
| rs7672929 | 4 | 158231766 | GLRB | intron | 0.465 | 0.596 | 0.404 | 0.433 |
| rs17035648 | 4 | 158237630 | GLRB | intron | 0.373 | 0.570 | 0.263 | 0.230 |
| rs4422461 | 4 | 158241732 | GLRB | intron | 0.371 | 0.557 | 0.262 | 0.256 |
| rs2880774 | 4 | 158247432 | GLRB | intron | 0.371 | 0.557 | 0.262 | 0.256 |
| rs11939934 | 4 | 158252859 | GLRB | intron | 0.454 | 0.347 | 0.405 | 0.409 |
| rs11947674 | 4 | 158258677 | GLRB | intron | 0.454 | 0.347 | 0.405 | 0.419 |
| rs3775723 | 4 | 158261402 | GLRB | intron | 0.371 | 0.557 | 0.262 | - |
| rs17035710 | 4 | 158268586 | GLRB | intron | 0.238 | 0.867 | 0.141 | 0.178 |
| rs17035723 | 4 | 158273864 | GLRB | intron | 0.373 | 0.570 | 0.263 | 0.256 |
| rs3775721 | 4 | 158283934 | GLRB | intron | 0.448 | 0.241 | 0.405 | 0.475 |
| rs11729510 | 4 | 158287030 | GLRB | intron | 0.356 | 0.267 | 0.257 | 0.244 |
| rs17035814 | 4 | 158294392 | GLRB | intron | 0.073 | 1.000 | 0.037 | 0.068 |
| rs17035818 | 4 | 158309162 | GLRB | intron | 0.048 | 1.000 | 0.024 | 0.022 |
| rs1129304 | 4 | 158311872 | GLRB | 3UTR | 0.467 | 0.899 | 0.383 | 0.411 |
| rs17035840 | 4 | 158322304 | GLRB | flanking_3UTR | 0.454 | 0.347 | 0.405 | 0.430 |
| rs11727838 | 4 | 158328046 | GLRB | flanking_3UTR | 0.349 | 0.099 | 0.263 | 0.267 |
| rs17035863 | 4 | 158332435 | LOC391707 | flanking_3UTR | 0.241 | 0.916 | 0.137 | 0.167 |
| rs17035876 | 4 | 158339702 | LOC391707 | flanking_3UTR | 0.257 | 1.000 | 0.151 | 0.156 |
| rs10025251 | 4 | 158351029 | LOC391707 | flanking_5UTR | 0.260 | 0.992 | 0.156 | 0.156 |
| rs10028901 | 4 | 158372969 | GRIA2 | intron | 0.257 | 0.938 | 0.154 | 0.148 |
| rs10011589 | 4 | 158377702 | GRIA2 | intron | 0.258 | 1.000 | 0.151 | 0.156 |
| rs9307959 | 4 | 158382470 | GRIA2 | intron | 0.270 | 0.926 | 0.163 | 0.167 |
| rs17035909 | 4 | 158388167 | GRIA2 | intron | 0.257 | 1.000 | 0.151 | 0.156 |
| rs6536225 | 4 | 158400771 | GRIA2 | intron | 0.279 | 1.000 | 0.165 | 0.167 |
| rs9993365 | 4 | 158405792 | GRIA2 | intron | 0.279 | 1.000 | 0.165 | 0.167 |
| rs17035920 | 4 | 158411349 | GRIA2 | intron | 0.279 | 1.000 | 0.165 | 0.170 |
| rs4418024 | 4 | 158417456 | GRIA2 | intron | 0.277 | 1.000 | 0.164 | 0.167 |
| rs10517665 | 4 | 158421852 | GRIA2 | intron | 0.259 | 0.907 | 0.149 | 0.159 |
| rs4538538 | 4 | 158427660 | GRIA2 | intron | 0.270 | 0.955 | 0.157 | 0.156 |
| rs10008950 | 4 | 158440684 | GRIA2 | intron | 0.277 | 1.000 | 0.164 | 0.167 |
| rs6536231 | 4 | 158445130 | GRIA2 | intron | 0.276 | 1.000 | 0.163 | 0.167 |
| rs10025086 | 4 | 158452435 | GRIA2 | intron | 0.267 | 1.000 | 0.156 | 0.156 |
| rs4302506 | 4 | 158458280 | GRIA2 | coding | 0.263 | 0.618 | 0.163 | 0.178 |
| rs4475186 | 4 | 158461990 | GRIA2 | intron | 0.267 | 0.668 | 0.165 | 0.189 |
| rs10007366 | 4 | 158476758 | GRIA2 | intron | 0.265 | 1.000 | 0.155 | 0.211 |
| rs7695870 | 4 | 158483226 | GRIA2 | intron | 0.263 | 1.000 | 0.154 | 0.205 |
| rs9683871 | 4 | 158490178 | GRIA2 | intron | 0.265 | 1.000 | 0.158 | 0.222 |
| rs6850942 | 4 | 158495007 | GRIA2 | intron | 0.265 | 1.000 | 0.158 | 0.227 |
| rs10012124 | 4 | 158499404 | GRIA2 | intron | 0.265 | 1.000 | 0.158 | - |
| rs4403097 | 4 | 158505047 | GRIA2 | 3UTR | 0.433 | 0.790 | 0.303 | 0.398 |
| rs11100101 | 4 | 158510013 | GRIA2 | flanking_3UTR | 0.431 | 0.809 | 0.302 | 0.389 |
| rs12643466 | 4 | 158515278 | GRIA2 | flanking_3UTR | 0.428 | 0.957 | 0.304 | 0.362 |
| rs17036018 | 4 | 158520217 | GRIA2 | flanking_3UTR | 0.425 | 1.000 | 0.302 | 0.375 |
| rs4691396 | 4 | 158525561 | GRIA2 | flanking_3UTR | 0.409 | 0.962 | 0.291 | 0.367 |
| rs12645401 | 4 | 158535352 | GRIA2 | flanking_3UTR | 0.425 | 1.000 | 0.302 | 0.364 |
| rs11100103 | 4 | 158541972 | GRIA2 | flanking_3UTR | 0.438 | 1.000 | 0.324 | 0.388 |
| rs6823909 | 4 | 158547155 | GRIA2 | flanking_3UTR | 0.267 | 0.872 | 0.162 | 0.151 |
| rs4234911 | 4 | 158553096 | GRIA2 | flanking_3UTR | 0.271 | 0.502 | 0.152 | 0.222 |
| rs6821249 | 4 | 158560910 | GRIA2 | flanking_3UTR | 0.444 | 0.990 | 0.327 | 0.389 |
| rs9992749 | 4 | 158566328 | GRIA2 | flanking_3UTR | 0.271 | 1.000 | 0.161 | 0.222 |
| rs12186189 | 4 | 158573371 | GRIA2 | flanking_3UTR | 0.430 | 0.979 | 0.317 | 0.378 |
| rs10517668 | 4 | 158576853 | GRIA2 | flanking_3UTR | 0.267 | 1.000 | 0.159 | 0.222 |
| rs7656328 | 4 | 158579646 | GRIA2 | flanking_3UTR | 0.430 | 0.979 | 0.317 | 0.389 |
| rs7698998 | 4 | 158583809 | GRIA2 | flanking_3UTR | 0.436 | 0.965 | 0.326 | 0.378 |
| rs6843849 | 4 | 158588880 | GRIA2 | flanking_3UTR | 0.425 | 0.893 | 0.314 | 0.378 |
| rs6818692 | 4 | 158593849 | GRIA2 | flanking_3UTR | 0.251 | 0.615 | 0.154 | 0.156 |
| rs17036150 | 4 | 158598223 | GRIA2 | flanking_3UTR | 0.251 | 0.437 | 0.157 | 0.270 |
