# Supplementary material for: Maternal Transmission Effect of a PDGF-C SNP on Nonsyndromic Cleft Lip with or without Palate from a Chinese Population
Source: PLoS One. 2012 Sep 28;7(9):e46477. doi: 10.1371/journal.pone.0046477 (PMC3460900; doi:10.1371/journal.pone.0046477)
Supplement: Table S3 — TDT and PO-LRT analysis. (DOC) [file pone.0046477.s003.doc]

Table S3. TDT and PO-LRT analysis

| SNP Name | Position | Gene | TDT by FBAT | | | |  | PO-LRT | |
| --- | --- | --- | --- | --- | --- | --- | --- | --- | --- |
|  |  |  | Overtransmitted | T | U | FBAT-P value |  | PO-LRT p-value | *I*_(m)_ |
| D4S1644 | 141970903 |  | 195 | 85 | 78 | 0.9035 |  |  |  |
| D4S2998 | 145785642 |  | 166 | 26 | 15 | 0.6776 |  |  |  |
| D4S3021 | 155155280 |  | 230^$^ | 29 | 20 | 0.4894 |  |  |  |
| D4S1556 | 157673422 | hCG_1814936 | 156 | 64 | 48 | 0.0820 |  |  |  |
| D4S1589 | 158005552 |  | 206 | 78 | 72 | 0.7941 |  |  |  |
| rs894588 | 158036593 | PDGF-C | A | 71 | 65 | 0.6069 |  | 0.0824 | 1.7461 |
| rs6851803 | 158076643 | PDGF-C | G | 14 | 5 | 0.0389 |  | 0.7542 | 1.2083 |
| rs17035464 | 158099171 | PDGF-C | A | 34 | 23 | 0.1451 |  | **0.0018** | 2.4603 |
| rs6845322 | 158103555 | PDGF-C | A | 86 | 69 | 0.1721 |  | 0.4275 | 1.0893 |
| D4S1498 | 158114042 | PDGF-C | 259 | 39 | 30 | 0.4498 |  |  |  |
| rs1443235 | 158116000 | PDGF-C | A | 67 | 66 | 0.9309 |  | 0.0485 | 0.4238 |
| rs13117461 | 158123643 | PDGF-C | G | 66 | 60 | 0.5930 |  | 0.2536 | 0.6523 |
| rs7674099 | 158131475 | PDGF-C | G | 76 | 71 | 0.6801 |  | 0.0186 | 0.3434 |
| rs1443230 | 158140930 | PDGF-C | G | 78 | 75 | 0.8084 |  | 0.0314 | 0.4059 |
| rs11945782 | 158144404 | PDGF-C | A | 67 | 56 | 0.3213 |  | 0.0188 | 0.4124 |
| rs716680 | 158151138 | PDGF-C | A | 77 | 74 | 0.8071 |  | 0.0394 | 0.401 |
| rs12649197 | 158155473 | PDGF-C | G | 77 | 75 | 0.8711 |  | 0.0279 | 0.3842 |
| rs765985 | 158159671 | PDGF-C | G | 76 | 75 | 0.9351 |  | 0.0312 | 0.4027 |
| rs17035528 | 158162784 | PDGF-C | G | 65 | 57 | 0.4689 |  | 0.0406 | 0.4772 |
| rs4535377 | 158169250 | GLRB | A | 74 | 68 | 0.6146 |  | 0.0436 | 0.55 |
| rs7668059 | 158175791 | GLRB | G | 74 | 70 | 0.7389 |  | 0.0398 | 0.4921 |
| rs6830495 | 158182104 | GLRB | G | 75 | 68 | 0.5583 |  | 0.1149 | 0.6907 |
| rs11945439 | 158188299 | GLRB | A | 63 | 51 | 0.2611 |  | 0.0596 | 0.6568 |
| rs17035590 | 158195266 | GLRB | G | 62 | 51 | 0.3008 |  | 0.0424 | 0.5351 |
| rs7689138 | 158207179 | GLRB | A | 37 | 27 | 0.2113 |  | 0.3286 | 1.7027 |
| rs7696725 | 158210676 | GLRB | G | 61 | 52 | 0.3972 |  | 0.0433 | 0.5709 |
| rs4690879 | 158215162 | GLRB | G | 68 | 61 | 0.5377 |  | 0.1027 | 0.6508 |
| rs6852066 | 158222346 | GLRB | A | 75 | 63 | 0.3070 |  | 0.1239 | 0.5289 |
| rs4615228 | 158226703 | GLRB | A | 60 | 57 | 0.7815 |  | 0.3380 | 1.5528 |
| rs7672929 | 158231766 | GLRB | A | 82 | 62 | 0.0956 |  | 0.1606 | 0.5888 |
| rs17035648 | 158237630 | GLRB | G | 60 | 57 | 0.7815 |  | 0.1314 | 0.4972 |
| rs4422461 | 158241732 | GLRB | A | 60 | 57 | 0.7815 |  | 0.1649 | 0.5551 |
| rs2880774 | 158247432 | GLRB | A | 60 | 57 | 0.7815 |  | 0.1649 | 0.5551 |
| rs11939934 | 158252859 | GLRB | A | 82 | 60 | 0.0649 |  | 0.3728 | 0.8295 |
| rs11947674 | 158258677 | GLRB | A | 82 | 60 | 0.0649 |  | 0.3728 | 0.8295 |
| rs3775723 | 158261402 | GLRB | G | 60 | 57 | 0.7815 |  | 0.1649 | 0.5551 |
| rs17035710 | 158268586 | GLRB | A | 47 | 27 | 0.0201 |  | 0.4823 | 1.2418 |
| rs17035723 | 158273864 | GLRB | A | 60 | 56 | 0.7103 |  | 0.1685 | 0.5573 |
| rs3775721 | 158283934 | GLRB | C | 82 | 58 | 0.0425 |  | 0.4191 | 0.7988 |
| rs11729510 | 158287030 | GLRB | G | 59 | 53 | 0.5708 |  | 0.2441 | 0.6426 |
| rs17035814 | 158294392 | GLRB | A | 17 | 5 | 0.0105 |  | 0.1031 | 2.5368 |
| rs17035818 | 158309162 | GLRB | C | 9 | 5 | 0.2850 |  | 0.0863 | 3.5502 |
| rs1129304 | 158311872 | GLRB | T | 82 | 63 | 0.1146 |  | 0.2428 | 0.6253 |
| rs17035840 | 158322304 | GLRB | G | 79 | 61 | 0.1282 |  | 0.4826 | 0.8352 |
| rs11727838 | 158328046 | GLRB | A | 55 | 54 | 0.9237 |  | 0.1144 | 0.4775 |
| rs17035863 | 158332435 | LOC391707 | C | 48 | 27 | 0.0153 |  | 0.3748 | 1.3218 |
| rs17035876 | 158339702 | LOC391707 | A | 49 | 30 | 0.0325 |  | 0.2999 | 0.5793 |
| rs10025251 | 158351029 | LOC391707 | G | 49 | 31 | 0.0442 |  | 0.5230 | 0.6856 |
| rs10028901 | 158372969 | GRIA2 | G | 49 | 30 | 0.0325 |  | 0.4461 | 0.6473 |
| rs10011589 | 158377702 | GRIA2 | A | 49 | 30 | 0.0325 |  | 0.3848 | 0.6257 |
| rs9307959 | 158382470 | GRIA2 | A | 51 | 32 | 0.0370 |  | 0.2992 | 0.5672 |
| rs17035909 | 158388167 | GRIA2 | T | 50 | 29 | 0.0181 |  | 0.3676 | 0.6417 |
| rs6536225 | 158400771 | GRIA2 | T | 53 | 32 | 0.0227 |  | 0.4069 | 0.6247 |
| rs9993365 | 158405792 | GRIA2 | A | 53 | 33 | 0.0310 |  | 0.5425 | 0.7131 |
| rs17035920 | 158411349 | GRIA2 | A | 53 | 32 | 0.0227 |  | 0.4329 | 0.6418 |
| rs4418024 | 158417456 | GRIA2 | G | 53 | 32 | 0.0227 |  | 0.4421 | 0.6482 |
| rs10517665 | 158421852 | GRIA2 | C | 51 | 27 | 0.0066 |  | 0.4668 | 0.701 |
| rs4538538 | 158427660 | GRIA2 | A | 51 | 32 | 0.0370 |  | 0.5028 | 0.743 |
| rs10008950 | 158440684 | GRIA2 | A | 51 | 33 | 0.0495 |  | 0.4131 | 0.6357 |
| rs6536231 | 158445130 | GRIA2 | A | 52 | 33 | 0.0393 |  | 0.4468 | 0.6594 |
| rs10025086 | 158452435 | GRIA2 | A | 50 | 32 | 0.0468 |  | 0.4160 | 0.6821 |
| rs4302506 | 158458280 | GRIA2 | A | 50 | 31 | 0.0348 |  | 0.3552 | 0.6995 |
| rs4475186 | 158461990 | GRIA2 | G | 51 | 31 | 0.0272 |  | 0.4236 | 0.7527 |
| rs10007366 | 158476758 | GRIA2 | T | 43 | 39 | 0.6587 |  | 0.0148 | 1.4453 |
| rs7695870 | 158483226 | GRIA2 | G | 43 | 37 | 0.5023 |  | 0.0128 | 1.3953 |
| rs9683871 | 158490178 | GRIA2 | A | 45 | 37 | 0.3770 |  | 0.0142 | 1.4303 |
| rs6850942 | 158495007 | GRIA2 | G | 44 | 38 | 0.5076 |  | 0.0063 | 1.4995 |
| rs10012124 | 158499404 | GRIA2 | A | 44 | 38 | 0.5076 |  | 0.0063 | 1.4994 |
| rs4403097 | 158505047 | GRIA2 | A | 75 | 57 | 0.1172 |  | 0.0485 | 1.2652 |
| rs11100101 | 158510013 | GRIA2 | G | 75 | 57 | 0.1172 |  | 0.0527 | 1.1426 |
| rs12643466 | 158515278 | GRIA2 | G | 73 | 58 | 0.1900 |  | 0.0474 | 1.1662 |
| rs17036018 | 158520217 | GRIA2 | A | 73 | 57 | 0.1605 |  | 0.0587 | 1.2332 |
| rs4691396 | 158525561 | GRIA2 | G | 70 | 55 | 0.1797 |  | 0.0597 | 1.1673 |
| rs12645401 | 158535352 | GRIA2 | T | 73 | 57 | 0.1605 |  | 0.0587 | 1.2332 |
| rs11100103 | 158541972 | GRIA2 | G | 75 | 60 | 0.1967 |  | 0.0166 | 1.7623 |
| rs6823909 | 158547155 | GRIA2 | A | 51 | 31 | 0.0272 |  | 0.0741 | 1.9002 |
| rs4234911 | 158553096 | GRIA2 | A | 44 | 38 | 0.5076 |  | 0.0050 | 1.7813 |
| D4S1629 | 158556260 | GRIA2 | 147 | 102 | 90 | 0.5125 |  |  |  |
| rs6821249 | 158560910 | GRIA2 | G | 76 | 61 | 0.2000 |  | 0.0346 | 1.6077 |
| rs9992749 | 158566328 | GRIA2 | C | 44 | 39 | 0.5831 |  | 0.0065 | 1.7921 |
| D4S413 | 158572604 | GRIA2 | 297 | 41 | 23 | 0.0197 |  |  |  |
| rs12186189 | 158573371 | GRIA2 | A | 71 | 60 | 0.3365 |  | 0.0320 | 1.6118 |
| rs10517668 | 158576853 | GRIA2 | A | 43 | 40 | 0.7419 |  | 0.0058 | 1.7586 |
| rs7656328 | 158579646 | GRIA2 | A | 73 | 59 | 0.2230 |  | 0.0233 | 1.4173 |
| rs7698998 | 158583809 | GRIA2 | G | 75 | 58 | 0.1405 |  | 0.0291 | 1.5227 |
| rs6843849 | 158588880 | GRIA2 | A | 72 | 59 | 0.2560 |  | 0.0259 | 1.5151 |
| rs6818692 | 158593849 | GRIA2 | A | 48 | 29 | 0.0304 |  | 0.0282 | 2.6516 |
| rs17036150 | 158598223 | GRIA2 | G | 48 | 29 | 0.0304 |  | 0.0522 | 2.2096 |
| D4S1603 | 163999458 |  | 194 | 50 | 44 | 0.3797 |  |  |  |
| D4S2431 | 175057242 |  |  | 26 | 13 | 0.2274 |  |  |  |

$ combined allele, including 230,226,234,240

Bold values represent results yield a significant p-value from PO-LRT after multiple tests correction
