# Supplementary material for: Maternal Transmission Effect of a PDGF-C SNP on Nonsyndromic Cleft Lip with or without Palate from a Chinese Population
Source: PLoS One. 2012 Sep 28;7(9):e46477. doi: 10.1371/journal.pone.0046477 (PMC3460900; doi:10.1371/journal.pone.0046477)
Supplement: Table S4 — Genotypes of family 152. (DOC) [file pone.0046477.s004.doc]

Table S4. Genotypes of Family 152

| SNP Name | Position | Child | | Father | | Mother | |
| --- | --- | --- | --- | --- | --- | --- | --- |
| D4S1556 | 157673422 | 156 | 156 | 156 | 156 | 156 | 156 |
| D4S1589 | 158005552 | 204 | 204 | 204 | 210 | 204 | 204 |
| rs894588 | 158036593 | G | G | A | G | G | G |
| rs6851803 | 158076643 | C | G | G | G | C | G |
| rs17035464 | 158099171 | **A** | **A** | T | T | **A** | **A** |
| rs6845322 | 158103555 | **A** | **A** | G | G | **A** | **A** |
| D4S1498 | 158114042 | **245** | **245** | 257 | 257 | **245** | **245** |
| rs1443235 | 158116000 | A | A | A | A | A | A |
| rs13117461 | 158123643 | G | G | G | G | G | G |
| rs7674099 | 158131475 | A | G | A | G | A | A |
| rs1443230 | 158140930 | A | G | G | G | A | A |
| rs11945782 | 158144404 | A | C | A | C | A | A |
| rs716680 | 158151138 | A | G | A | A | G | G |
| rs12649197 | 158155473 | A | G | G | G | A | A |
| rs765985 | 158159671 | A | G | G | G | A | A |
| rs17035528 | 158162784 | A | G | A | A | G | G |
| rs4535377 | 158169250 | A | G | G | G | A | A |
| rs7668059 | 158175791 | A | G | A | A | G | G |
| rs6830495 | 158182104 | A | G | A | A | G | G |
| rs11945439 | 158188299 | A | C | C | C | A | A |
| rs17035590 | 158195266 | A | G | A | G | G | G |
| rs7689138 | 158207179 | C | C | A | C | C | C |
| rs7696725 | 158210676 | C | G | C | G | G | G |
| rs4690879 | 158215162 | A | G | A | G | G | G |
| rs6852066 | 158222346 | A | G | A | G | A | A |
| rs4615228 | 158226703 | A | G | G | G | A | A |
| rs7672929 | 158231766 | A | G | A | G | A | A |
| rs17035648 | 158237630 | A | G | A | A | G | G |
| rs4422461 | 158241732 | A | G | G | G | A | A |
| rs2880774 | 158247432 | A | G | G | G | A | A |
| rs11939934 | 158252859 | A | G | A | G | A | A |
| rs11947674 | 158258677 | A | C | A | C | A | A |
| rs3775723 | 158261402 | A | G | A | A | G | G |
| rs17035710 | 158268586 | T | T | A | T | T | T |
| rs17035723 | 158273864 | A | G | G | G | A | A |
| rs3775721 | 158283934 | A | C | A | C | C | C |
| rs11729510 | 158287030 | A | G | G | G | A | A |
| rs17035814 | 158294392 | G | G | A | G | G | G |
| rs17035818 | 158309162 | G | G | C | G | G | G |
| rs1129304 | 158311872 | A | T | A | T | T | T |
| rs17035840 | 158322304 | A | G | A | G | G | G |
| rs11727838 | 158328046 | A | G | A | A | A | G |
| rs17035863 | 158332435 | A | A | A | C | A | A |
| rs17035876 | 158339702 | G | G | G | G | G | G |
| rs10025251 | 158351029 | A | A | A | A | A | A |
| rs10028901 | 158372969 | C | C | C | C | C | C |
| rs10011589 | 158377702 | T | T | T | T | T | T |
| rs9307959 | 158382470 | G | G | G | G | G | G |
| rs17035909 | 158388167 | A | A | A | A | A | A |
| rs6536225 | 158400771 | A | A | A | A | A | A |
| rs9993365 | 158405792 | G | G | G | G | G | G |
| rs17035920 | 158411349 | G | G | G | G | G | G |
| rs4418024 | 158417456 | C | C | C | C | C | C |
| rs10517665 | 158421852 | G | G | G | G | G | G |
| rs4538538 | 158427660 | G | G | G | G | G | G |
| rs10008950 | 158440684 | G | G | G | G | G | G |
| rs6536231 | 158445130 | G | G | G | G | G | G |
| rs10025086 | 158452435 | T | T | T | T | T | T |
| rs4302506 | 158458280 | G | G | G | G | G | G |
| rs4475186 | 158461990 | A | A | A | A | A | A |
| rs10007366 | 158476758 | T | T | T | T | T | T |
| rs7695870 | 158483226 | G | G | G | G | G | G |
| rs9683871 | 158490178 | A | A | A | A | A | A |
| rs6850942 | 158495007 | G | G | G | G | G | G |
| rs10012124 | 158499404 | A | A | A | A | A | A |
| rs4403097 | 158505047 | G | G | G | G | G | G |
| rs11100101 | 158510013 | A | A | A | A | A | A |
| rs12643466 | 158515278 | A | A | A | A | A | A |
| rs17036018 | 158520217 | G | G | G | G | G | G |
| rs4691396 | 158525561 | A | A | A | A | A | A |
| rs12645401 | 158535352 | A | A | A | A | A | A |
| rs11100103 | 158541972 | A | A | A | A | A | G |
| rs6823909 | 158547155 | G | G | G | G | A | G |
| rs4234911 | 158553096 | A | A | A | A | A | A |
| D4S1629 | 158556260 | 147 | 151 | 151 | 155 | 139 | 147 |
| rs6821249 | 158560910 | A | A | A | A | A | G |
| rs9992749 | 158566328 | C | C | C | C | C | C |
| D4S413 | 158572604 | 285 | 285 | 285 | 285 | 285 | 293 |
| rs12186189 | 158573371 | C | C | C | C | A | C |
| rs10517668 | 158576853 | A | A | A | A | A | A |
| rs7656328 | 158579646 | G | G | G | G | A | G |
| rs7698998 | 158583809 | A | A | A | A | A | G |
| rs6843849 | 158588880 | G | G | G | G | A | G |
| rs6818692 | 158593849 | T | T | T | T | A | T |
| rs17036150 | 158598223 | A | A | A | A | A | G |
| D4S1603 | 163999458 | 194 | 196 | 192 | 194 | 194 | 196 |
| D4S2431 | 175057242 | 240 | 240 | 240 | 244 | 240 | 240 |
