# Supplementary material for: Maternal Transmission Effect of a PDGF-C SNP on Nonsyndromic Cleft Lip with or without Palate from a Chinese Population
Source: PLoS One. 2012 Sep 28;7(9):e46477. doi: 10.1371/journal.pone.0046477 (PMC3460900; doi:10.1371/journal.pone.0046477)
Supplement: Table S5 — Haplotype TDT Analysis. (DOC) [file pone.0046477.s005.doc]

Table S5. Haplotype TDT Analysis

| Block # | Block | Freq. | Chi Square | P Value | Permutation p-value |
| --- | --- | --- | --- | --- | --- |
| Block 1 | AA | 0.549 | 1.853 | 0.1734 | 0.9807 |
| Block 1 | AG | 0.347 | 0.263 | 0.6082 | 1 |
| Block 1 | TG | 0.105 | 2.123 | 0.1451 | 0.8522 |
| Block 2 | GA | 0.613 | 0.169 | 0.6806 | 1 |
| Block 2 | CG | 0.286 | 0.29 | 0.5902 | 1 |
| Block 2 | GG | 0.098 | 2.281 | 0.131 | 0.816 |
| Block 3 | AAGAAGAGGAG | 0.649 | 0 | 0.9997 | 1 |
| Block 3 | GCAGGAGAACA | 0.203 | 1.11 | 0.2921 | 0.995 |
| Block 3 | GAAGGGGAAAG | 0.085 | 1.723 | 0.1893 | 0.9817 |
| Block 3 | GCAGGAAGGAG | 0.021 | 1.923 | 0.1655 | 0.9794 |
| Block 3 | GAAGGAGAACG | 0.014 | 0.5 | 0.4795 | 1 |
| Block 4 | GGGAGGGCATGA | 0.591 | 3.128 | 0.077 | 0.6783 |
| Block 4 | AAAGAAAAGTAC | 0.259 | 0.138 | 0.7103 | 1 |
| Block 4 | AGAAGGAAAAGC | 0.123 | 2.25 | 0.1336 | 0.8234 |
| Block 4 | GGAAGGAAAAGC | 0.013 | 7.999 | 0.0047 | 0.0645 |
| Block 5 | AA | 0.597 | 2.597 | 0.1071 | 0.7695 |
| Block 5 | TG | 0.38 | 2.282 | 0.1309 | 0.8137 |
| Block 5 | AG | 0.022 | 0.077 | 0.7815 | 1 |
| Block 6 | AA | 0.601 | 2.797 | 0.0944 | 0.7447 |
| Block 6 | GA | 0.265 | 0.009 | 0.924 | 1 |
| Block 6 | AC | 0.134 | 6.041 | 0.014 | 0.1914 |
| Block 7 | GACTGAAGGCGGGGTGATGAGA | 0.668 | 1.216 | 0.2702 | 0.9931 |
| Block 7 | GACTGAAGGCGGGGTGAAAGAG | 0.152 | 0.2 | 0.6547 | 1 |
| Block 7 | AGGAATTAAGCAAAAAGTGAGA | 0.144 | 4.701 | 0.0302 | 0.3172 |
| Block 8 | GAAG | 0.693 | 1.939 | 0.1637 | 0.9693 |
| Block 8 | AGGA | 0.3 | 2.492 | 0.1144 | 0.7748 |
| Block 9 | AAAGAACCAGAG | 0.657 | 1.614 | 0.2039 | 0.9845 |
| Block 9 | GTGGGGAAGAGA | 0.158 | 0.2 | 0.6547 | 1 |
| Block 9 | GTGAAGCAAAGA | 0.129 | 8.138 | 0.0043 | 0.0612 |
| Block 9 | AAGAAGCAAAGA | 0.022 | 0.286 | 0.593 | 1 |
| Block 9 | AAAGAACCAGGG | 0.013 | 0.489 | 0.4842 | 1 |
| Block 10 | TA | 0.845 | 4.688 | 0.0304 | 0.3265 |
| Block 10 | AG | 0.153 | 5.263 | 0.0218 | 0.2472 |
