# Supplementary figures and images for: Maternal Transmission Effect of a PDGF-C SNP on Nonsyndromic Cleft Lip with or without Palate from a Chinese Population
Source: PLoS One. 2012 Sep 28;7(9):e46477. doi: 10.1371/journal.pone.0046477 (PMC3460900; doi:10.1371/journal.pone.0046477)

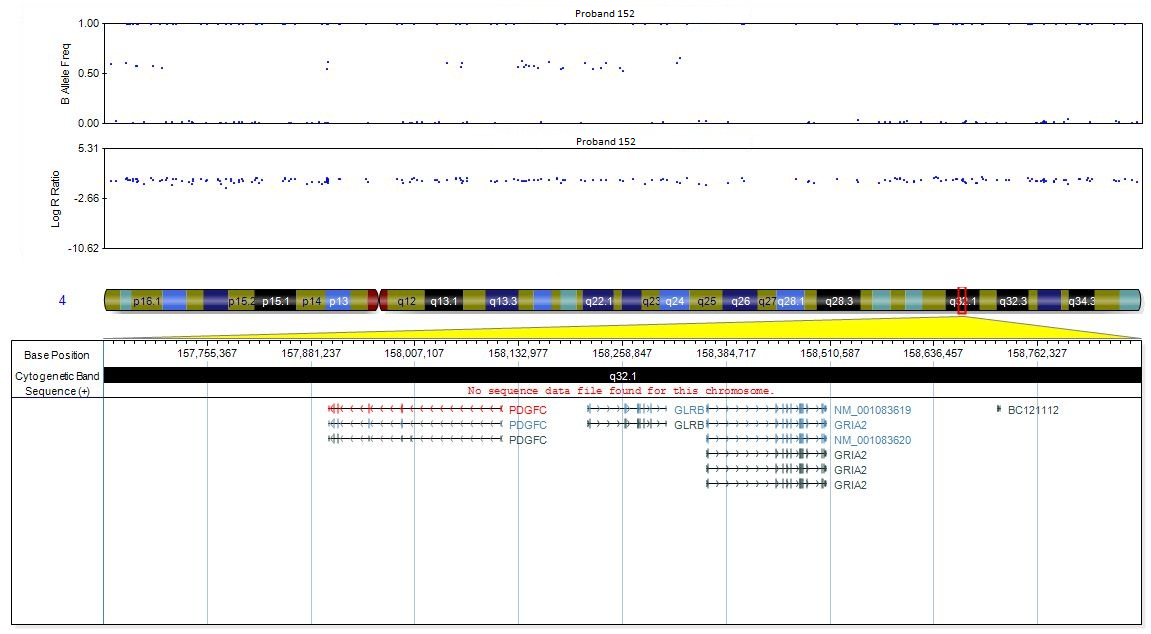

Supplement: Figure S1 — CNV analysis of proband 152 on 4q32. The whole genome genotyping and CNV analysis was performed by Illumina Human 660W-Quad v1 DNA Analysis BeadChip. (TIF) [file pone.0046477.s006.tif]
